# 

**Published:** 1902-11-29

**Authors:** 


### NOTICE TO CORRESPONDENTS.

**All MSS., Letters, Books for Review, and other matters intended for the Editor should be addressed The Editor, "The Hospital," The Hospital Building, 28 & 29 Southampton Street, Strand, W.C.**

**The Editor cannot undertake to return rejected MS., even when accompanied by stamped directed envelope.**

**Notice to Advertisers and Subscribers.**

**All Advertisements, Orders for Copies of The Hospital, and Business Communications should be addressed to, THE MANAGER, (Not to the Editor),** The Hospital, 28 & 29 Southampton Street, Strand, London, W.C.

The Cost of Subscription, post free, is as follows.—Per week, 2½d.; per quarter, 2s. 8d.; per half-year, 5s. 3d.; per annum, 10s. 6d. Foreign:—Per annum, 15s. 2d., payable, in all cases, in advance.

**NOTICE.—Vol. XXXII. of "The Hospital" (April to September 1902) in handsome cloth binding is now on sale at the office of this Journal, price, 7s. 6d. Cases for binding the Numbers contained in this Volume 1s. 6d. Index to Vol. XXXII., 2½d., post free.**

**The Monthly Part for December will be ready on December 1. Price 6d., by post 8d.**

### Scraps and Gleanings.

His Majesty the King has sent a present of venison for the patients at the Metropolitan Hospital, Kingsland Road.

There are at present only 14 small-pox patients under treatment in London

In the village of Chalfont, St. Giles, a concert has been organised by a member of the parish council in aid of a fund to pay for lighting the village, and so saving the villagers this expense, which they at present have to provide. The first concert realised £12, and similar entertainments are to be held periodically with the same object.

